# Magnetic-Field-Inspired Navigation for Robots in Complex and Unknown Environments

**DOI:** 10.3389/frobt.2022.834177

**Published:** 2022-02-18

**Authors:** Ahmad Ataka, Hak-Keung Lam, Kaspar Althoefer

**Affiliations:** ^1^ Signal Processing Laboratory, Department of Electrical and Information Engineering, Universitas Gadjah Mada, Yogyakarta, Indonesia; ^2^ The Centre for Robotics Research (CoRe), Department of Engineering, King’s College London, London, United Kingdom; ^3^ The Centre for Advanced Robotics @ Queen Mary (ARQ), Faculty of Science and Engineering, Queen Mary University of London, London, United Kingdom

**Keywords:** magnetic-field-inspired navigation, reactive navigation, motion control, path planning for manipulators, obstacle advoidance

## Abstract

Over the course of the past decade, we have witnessed a huge expansion in robotic applications, most notably from well-defined industrial environments into considerably more complex environments. The obstacles that these environments often contain present robotics with a new challenge - to equip robots with a real-time capability of avoiding them. In this paper, we propose a magnetic-field-inspired navigation method that significantly has several advantages over alternative systems. Most importantly, *1*) it guarantees obstacle avoidance for both convex and non-convex obstacles, *2*) goal convergence is still guaranteed for point-like robots in environments with convex obstacles and non-maze concave obstacles, *3*) no prior knowledge of the environment, such as the position and geometry of the obstacles, is needed, *4*) it only requires temporally and spatially local environmental sensor information, and *5*) it can be implemented on a wide range of robotic platforms in both 2D and 3D environments. The proposed navigation algorithm is validated in simulation scenarios as well as through experimentation. The results demonstrate that robotic platforms, ranging from planar point-like robots to robot arm structures such as the Baxter robot, can successfully navigate toward desired targets within an obstacle-laden environment.

## 1 Introduction

Over the past decade, robotic technologies have found their way into an ever-growing number of fields. No longer limited to the well defined and structured environments commonly found in industrial sites, robotic devices have moved into all manner of environments as they become key “players” in a variety of domestic and care tasks, as well as in post-disaster evacuation and nuclear decommissioning. In line with this shift in robotic applications, the need to equip a robot with the ability to navigate arbitrarily-shaped obstacles within unknown environments, while relying on limited sensor information, becomes increasingly important.

The problems of robot navigation, path planning, and obstacle avoidance have been widely explored over the last 30 years (Latombe, 1991), (Choset, 2005). In the early years, solutions focused on producing geometrical paths for the robot to take to reach its target. While theoretically elegant, this type of planning was found to be rather impractical due to the costly numerical computation that was needed to create the configuration space (C-Space) (LaValle, 2011). This prompted a new approach, known as sampling-based planning which randomly samples and checks whether a configuration lies in free space or not (Choset, 2005). Other algorithms produce initial paths for static environments that are based on a known map and modify those paths online (Brock and Khatib, 2002; Vannoy and Xiao, 2008). Another approach that employs potential function with a single global minimum has been proposed. Examples of this kind of navigation method include a harmonic function (Garrido et al., 2010) and a fast-marching method (Valero-Gomez et al., 2013). The properties of magnetic fields have also inspired several researchers to create navigation systems that mimic the behaviour of magnetic fields. Examples include those reported in (Singh et al., 1996, 1997) and (Haddadin et al., 2011). However, and significantly, most of these algorithms rely on perfect knowledge of the environment prior to the onset of any motion on the part of the robotic device.

For applications in which no prior knowledge of the environment is available, a reactive sensor-based navigation system is employed, in which the robot senses the environment and the acquired sensor information is used to produce motion signals in real-time (Valle, 2011). An example of widely-used reactive navigation is the Artificial Potential Field (APF) method in which obstacles produce a repulsive behaviour repelling the robot while the target attracts it (Khatib, 1985). The main advantage of this method is its simplicity which indeed has led to its widespread application (Choset, 2005) and to the emergence of a variety of similar methods (Park et al., 2008). These methods, however, all suffer from the well-documented problem of local-minima often causing the robot to get stuck in undesired configurations and fail to reach its target (Choset, 2005). To avoid this problem without diminishing its reactivity, a vortex field, designed to circulate around the obstacle surface, is incorporated (Rizqi et al., 2014), although in this case, the authors present no guarantees in terms of stability or collision avoidance capability. Inspiration from computational geometry has also led to the use of power diagrams as a route to solving the problem of reactive navigation in an unknown environment (Arslan and Koditschek, 2016). Despite being able to guarantee obstacle avoidance and target convergence, this approach is however only suitable for a topologically simple environment consisting of spherical obstacles in a planar world.

“Circular fields” or “gyroscopic forces” (Singh et al., 1996, 1997; Haddadin et al., 2011), which work by redirecting rather than repelling the robot, have been researched extensively during the last decade and an algorithm based on this has been used in a planar point-like robot to achieve tasks such as boundary following (Zhang et al., 2004a,b), obstacle avoidance (Chang and Marsden, 2003), and formation control for multi-agent systems (Chang and Marsden, 2003). However, obstacle avoidance and goal convergence are only guaranteed in environments with convex obstacles, limiting its applications in realistic environments. Applying these techniques for non-convex obstacles is not straightforward because simply redirecting the direction of the robot’s movement to follow non-convex obstacles’ surface is not sufficient to guarantee collision avoidance. Adding a repulsive term will help to guarantee collision avoidance. However, it affects the goal convergence and potentially leads the robot towards a local minimum. More recent work has explored the possibility of using a gyroscopic control method for maze-like environments (Matveev et al., 2013) as well as environments with dynamic obstacles (Savkin and Wang, 2013, 2014) and those with deforming obstacles (Matveev et al., 2012, 2015). These methods, summarized in (Savkin and Wang, 2015), were however all specifically designed for planar unicycle-like mobile robots. Subsequent to that, efforts have been focused on the application of gyroscopic forces in fully-actuated or under-actuated robotics systems in 3D environments (Garimella et al., 2016). This method, though, is specifically designed for environments consisting of spherical and cylindrical obstacles. While efforts to apply gyroscopic force algorithms to formation control of multiple robots in 3D have also been reported (Justh and Krishnaprasad, 2004; Sabattini et al., 2013, 2017), these algorithms only provide solutions for collision avoidance in point-like robots.

In this paper, we present a reactive magnetic-field-inspired navigation system for robots in 3D environments. The moving robot generates an artificially-induced electric current on the surface of an obstacle which, in turn, induces a magnetic field on the robot. Although reliant on local sensor information only, indeed without any required knowledge of the geometry or position of any obstacle in the given environment, the proposed algorithm has the ability to navigate point-like robots to their desired targets without any local minima issues. Our algorithm outperforms other magnetic-field-inspired navigation algorithms (Singh et al., 1996, 1997; Haddadin et al., 2011) as no requisite knowledge of the environment is required. It is also superior to the reactive gyroscopic force methods (Zhang et al., 2004a,b; Chang and Marsden, 2003; Garimella et al., 2016; Justh and Krishnaprasad, 2004; Sabattini et al., 2013, 2017), indeed including our own recent works (Ataka et al., 2018a,b), as obstacle avoidance and goal convergence are guaranteed in environments consisting of convex and non-maze concave obstacles. Moreover, our algorithm is not limited to 2D environments, as is the case for methods presented in (Savkin and Wang, 2015), but also enables the guidance of robots to their target within 3D environments. Alongside these advantages, our algorithm has a further benefit, which relates to its generic nature - and its consequential applicability to a variety of robotic platforms operating in a wide range of settings. To the best of our knowledge, we believe that this paper is the first to propose a reactive, magnetic-field based robot navigation system that is capable of guiding robots around arbitrary-shaped and unknown convex and non-maze concave obstacles to its target, within 3D environments.

## 2 Inspiration From Nature

**FIGURE 1:**
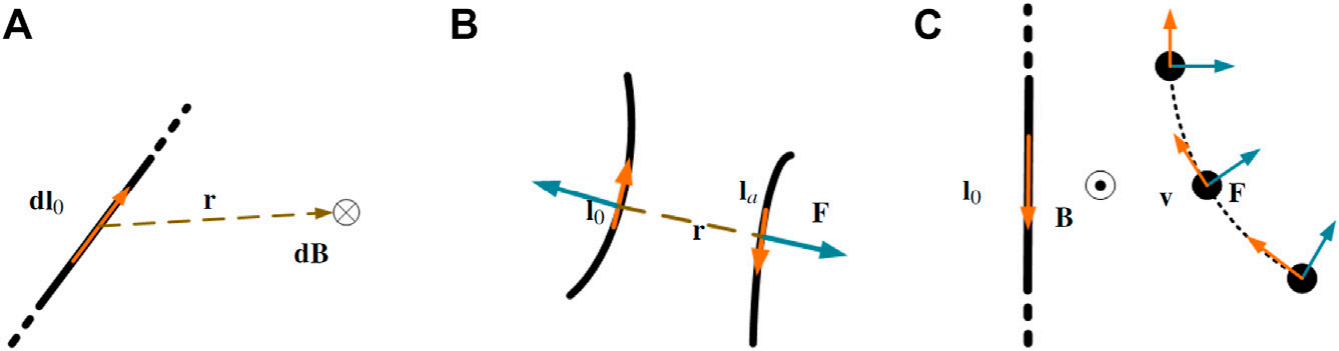
**(A)** A current-carrying wire with electric current in the direction of **dl**_*o*_ will produce magnetic field **dB** in the space around it. In this configuration, **dB** points inside the paper. **(B)** Two wires with electric currents **l**_*a*_ and **l**_*o*_, each flowing towards opposite direction of each other, will generate a repulsive force **F** on both wires directed away from each other. **(C)** A positively-charged particle is affected by Lorentz force **F** due to the presence of magnetic field **B** induced by current **l**_*o*_.

Our ideas are inspired by phenomena observed in classical electromagnetism. Prominent among its underlying theorems is the Biot-Savart law which describes the relationship between a current-carrying conductor and the surrounding magnetic field that it produces. A current-carrying wire segment with an infinitesimal length **dl**_*o*_ and electrical current *i*_*o*_ will induce a magnetic field **dB**. This magnetic field, whose direction is illustrated in Figure 1A, is expressed by the following equation (Halliday et al., 2013)

$$dB=\frac{\mu_{0}}{4\pi }\frac{i_{o}{dl}_{o}\times r}{|r{|}^{3}},$$ (1)

Where **r** specifies the position of an arbitrary point in the surrounding space of the wire segment relative to the current-carrying wire, *μ*_0_ specifies a permeability constant, while × specifies the operation of vector cross product. The total magnetic field produced by the wire can be derived by integrating the above operation over a wire length *l* which will depend on the wire configuration.

This magnetic field will produce a force **dF** on any other current-carrying wire with an infinitesimal length **dl**_*a*_ and current *i*_*a*_ flowing in the opposite direction, as illustrated in Figure 1B, and expressed by the following equation

$$dF=i_{a}{dl}_{a}\times B.$$ (2)

The direction of the force is perpendicular to the direction of both the electric current *i*_*a*_ (in the direction of the vector **dl**_*a*_) and magnetic field vector **B**. This force also generates a repulsive behaviour in the pair of wires, such that it drives the wires apart.

In a similar manner, as illustrated in Figure 1C, the movement of a charged particle *q* will be influenced by the magnetic field as it moves into the vicinity of the current-carrying wire. This magnetic field will apply a force **F** to the moving particle in a direction perpendicular to the particle’s velocity vector **v** and the magnetic field vector **B**. This force, usually known as Lorentz force, is expressed by the following equation (Halliday et al., 2013)

F=qv×B. (3)

Substituting the expression for **B** from (1) and abandoning the infinitesimal notation, the force both in Eqs 2, 3 can be expressed as

$$F=\frac{\mu_{0}qi_{o}}{4\pi }\;\frac{l_{a}\times \left(l_{o}\times r\right)}{|r{|}^{3}},$$ (4)

Where **l**_*a*_ denotes either the direction of the current in the case of the wire in Figure 1B or the particle’s velocity in the case of the charged particle in Figure 1C.

As shown in Figure 1C, the interaction between the magnetic field and the moving charged particle produces a force whose direction is perpendicular to that of the particle’s motion. The net result is that the particle’s direction of movement is altered, rather than its velocity decreased. Taking inspiration from this behaviour pattern, a robotic arm can be envisaged as a moving charged particle whose velocity is expressed by vector **v** while the surface of a potential obstacle can be thought of as a current-carrying wire. Collision avoidance can then be achieved if the robot induces an artificial current **l**_*o*_ that flows over the surface of an obstacle expressed by position vector **r**_*o*_ (with respect to the robot’s own position). This artificial current will then apply a force **F** onto the robot, pushing the robot away from and around the surface of any obstacles in its pathway.

Using our definition of the positional vector, we get **r**_*o*_ = − **r**. Thus, we can rewrite the Eq. 4 in the following form

$$F=c\;l_{a}\times \left(r_{o}\times l_{o}\right)\;f\left(\left|r_{o}|,|\dot{p}\right|\right),$$ (5)

In which *c* > 0 is a positive constant, **l**_*a*_ is defined as the direction of the robot’s velocity vector, while $f\left(\left|r_{o}|,|\dot{p}\right|\right)\geq 0$ is defined as a function which is dependent upon the distance between the obstacle and the robot |**r**_*o*_| and/or speed of the robot $\left|\dot{p}\right|$ . To help highlight the key characteristics of the algorithm, a skew-symmetric matrix $\hat{l}$ is used to replace the operation of vector cross product **l**× of an arbitrary vector $l={\left[\begin{array}{ccc} l_{x} & l_{y} & l_{z} \end{array}\right]}^{T}$ . This matrix is defined as

$$\hat{l}=\left[\begin{array}{ccc} 0 & -l_{z} & l_{y}\\ l_{z} & 0 & -l_{x}\\ -l_{y} & l_{x} & 0 \end{array}\right].$$ (6)

We can design this artificial vector field to produce a desired robot behaviour by defining the current direction **l**_*o*_ on the obstacle surface and the scalar function $f\left(\left|r_{o}|,|\dot{p}\right|\right)$ as per the explanation in Section 4.

## 3 Problem Formulation

Let us consider a point-mass robot in a bounded work-space $W\subset R^{2}$ or $W\subset R^{3}$ whose position is described by a position vector p∈W . The robot is assumed to be equipped with a local sensor that is sensitive to the surrounding environment within a sphere of radius *r*_*s*_ centred at the robot’s position **p**. The robot is also able to measure, in real-time, its velocity $\dot{p}$ , whose direction is defined as

$$l_{a}=\frac{\dot{p}}{\left|\dot{p}\right|}.$$ (7)

Lastly, the robot’s actuators are assumed to be non-saturatable, i.e. they will always have the necessary energy to produce the required movement. Significantly, the environment is deemed completely unknown to the robot prior to its actuation.

**FIGURE 2:**
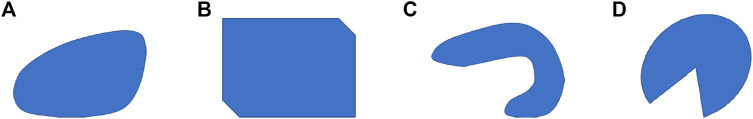
The environment considered in the paper can consists of any of the following obstacles: **(A)** a smooth convex obstacle, **(B)** a non-smooth convex obstacle, **(C)** a smooth concave obstacle, and **(D)** a non-smooth concave obstacle.

We assume that the environment of the robot consists of m∈N number of fixed obstacles $O_{i}$ . The obstacle $O_{i}$ can be any of the following types:

1. a member of a convex set with smooth boundary, such as illustrated in Figure 2A,
2. a member of a convex set with non-smooth boundary, such as illustrated in Figure 2B,
3. a member of a *simple* concave set with smooth boundary, such as illustrated in Figure 2C,
4. a member of a *simple* concave set with non-smooth boundary, such as illustrated in Figure 2D.

The term *simple* refers to non-maze geometry in which the obstacle surface will not require the robot to change its direction of motion more than 180° from its initial trajectory towards the target while following the obstacle boundary. The free space is then formally defined as $F=W\backslash \overset{m}{\underset{i=1}{⋃}}O_{i}$ . We assume that the robot starts moving from an initial position $p_{s}\in F$ to the desired target position $p_{g}\in F$ located at some distance from any of the surfaces of an obstacle. Assuming a double integrator system with dynamics described as

$$\ddot{p}=u,$$ (8)

We want to determine the control signal **u** which will guide the robot’s position **p**(*t*) towards the desired position **p**_**g**_ as *t* → *∞* while keeping the robot free from collision throughout its entire route, which is formally defined as

$$p\left(t\right)\in F,\forall t.$$ (9)

## 4 Proposed Algorithm

### 4.1 General Algorithm

To ensure collision avoidance while moving toward the target, we introduce a magnetic-field-inspired vector field **F**_**o**_ consisting of two terms: a boundary-following vector field **F**_**b**_ and a collision-avoidance vector field **F**_**a**_ as follows

$$F_{o}=F_{b}+F_{a}.$$ (10)

The magnetic-field-inspired vector fields **F**_**b**_ and **F**_**a**_ are created by artificial currents on the obstacle surface induced by the moving robot, **l**_*o*_ and **l**_*o*⊥_ respectively.

To induce boundary-following behaviour, so that the robot follows the direction of artificial current **l**_*o*_, the vector field **F**_**b**_ is defined as

$$F_{b}=c\;l_{a}\times \left(l_{o}\times l_{a}\right)\;f\left(\left|r_{o}|,|\dot{p}\right|\right).$$ (11)

The behaviour of the moving robot under the influence of the vector field **F**_**b**_ mimics the behaviour of a charged particle moving in the proximity of a current-carrying wire as illustrated in Figure 1C.

**FIGURE 3:**
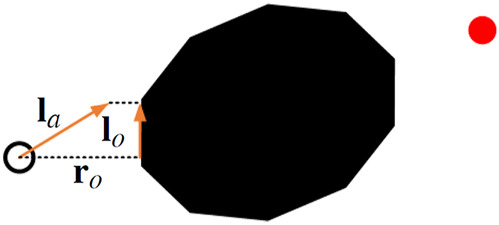
The workspace of a robot on its way towards the target in the close proximity of the polygonal obstacle. The artificial current on the surface of obstacle **l**_*o*_ is designed to have the same direction as the projection of the robot’s velocity **l**_*a*_ on to the obstacle surface.

In order to generate an artificial current **l**_*o*_ on the surface of an obstacle, let us start with a point-mass robot in $R^{2}$ moving in an environment as illustrated in Figure 3.

In this planar environment, the current on the obstacle surface needs to be induced in such a way that the robot safely circumvents the obstacle by following the obstacle boundary in either an anti-clockwise or a clockwise direction. At the same time, the effect of the current should not dramatically alter the path that the robot is inclined to take to reach its target - it simply needs to ensure that collisions are avoided and unnecessary oscillations are mitigated. To satisfy this requirement, we define the proposed artificial current direction **l**_*o*_ as a projection of the robot’s velocity direction **l**_*a*_ on to the surface of a nearby obstacle, as illustrated in Figure 3.

Despite having no knowledge of the obstacle’s geometry, we can still make a practical assumption regarding the surface of a nearby obstacle using local sensory information only.

Suppose that the robot senses the closest point of the obstacle surface located at position **r**_*o*_ relative to the robot’s current position. The surface of the obstacle at this specific point is then assumed to have a normal vector **n**_*o*_ in the opposite direction of **r**_*o*_, i.e. the surface is assumed to be perpendicular to the vector **r**_*o*_. The artificial current **l**_*o*_ can therefore be described as:

$$l_{o}=l_{a}-\frac{\left(l_{a}^{T}r_{o}\right)r_{o}}{|r_{o}{|}^{2}}.$$ (12)

Finally, to complete the definition of the boundary-following vector field **F**_**b**_, the scalar function $f\left(\left|r_{o}|,|\dot{p}\right|\right)$ is chosen as:

$$f\left(\left|r_{o}|,|\dot{p}\right|\right)=\frac{\left|\dot{p}\right|}{\left|r_{o}\right|}.$$ (13)

To keep the robot at a safe distance from the obstacle surface, the vector field **F**_**a**_ behaves much in the same way as a pair of current-carrying wires in which the current flowing in one wire is in the opposite direction to that of the other - *see* Section 2 (Figure 1B). The robot moving in a direction of **l**_*a*_ will induce an artificial current **l**_*o*⊥_ in the opposite direction of current **l**_*o*_ described in (12) as follows:

$$l_{o⊥}=-\left(l_{a}-\frac{\left(l_{a}^{T}r_{o}\right)r_{o}}{|r_{o}{|}^{2}}\right).$$ (14)

The field equation **F**_**a**_ can then be derived following the general equation in (5) with the scalar function $f\left(\left|r_{o}|,|\dot{p}\right|\right)$ chosen to be inversely proportional to the robot-obstacle distance *r* as follows

$$F_{a}=l_{a}\times \left(\frac{r_{o}}{r}\times l_{o⊥}\right)\;\frac{c_{⊥}}{r},$$ (15)

Where *c*_⊥_ denotes a positive constant.

### 4.2 Properties of the Proposed Algorithm

In the following section, we outline the key properties of our navigation algorithm. To simplify the analysis, we describe the properties inherently possessed by each of the two vector field components: the boundary-following vector field **F**_**b**_ and the collision-avoidance vector field **F**_**a**_.

Several properties of the boundary-following vector field **F**_**b**_, outlined in previous work by the authors (Ataka et al., 2018a), can be summarised in Lemma 1 to Lemma 4. Please refer to our previous work in (Ataka et al., 2018a) for the proofs of these lemmas.

Lemma 1. *The force* **F**_**b**_ *described in* (11) *does not affect the speed of the robot* $v=|\dot{p}|$ *.*

Lemma 2. S*uppose that the robot is in the vicinity of a flat obstacle surface and it is located far enough from the obstacle such that no collision can occur during its movement. The force* **F**_**b**_ *will guide the robot to reach a direction of the artificial current* **l**_*o*_ *, causing the robot’s direction to be parallel to the obstacle surface.*

Lemma 3. *Suppose that the closest surface of a convex-shaped obstacle is located at initial distance* *r*_0_ = |**r**_*o*_| *from the robot. The robot will never touch the surface of the obstacle as long as its initial direction* **l**_*a*_ *is not in line with the direction of vector* **r**_*o*_ *connecting the robot and the obstacle.*

Lemma 4. T*he robot*’*s direction of movement will asymptotically reach a direction in line with the direction of the artificial current. This equilibrium direction is globally asymptotically stable.*

Lemma 3–4 show that the boundary-following vector field **F**_**b**_ ensures that the robot follows a direction parallel to the nearby obstacle surface and hence avoids collision with a convex obstacle. However, these features do not directly control the distance to the obstacle surface. This creates a problem for environments with concave obstacles as maintaining the robot’s direction alone is not sufficient to avoid collisions. It is here that the collision-avoidance vector field **F**_**a**_ comes into play. The properties of this vector field are as follows:

Lemma 5. *The force* **F**_**a**_ *in* (15) *will not change the magnitude of robot*’*s velocity* $v=|\dot{p}|$ *.*

Proof. We introduce vector $a_{a}=\left(\frac{r_{o}}{r}\times l_{o⊥}\right)\;\frac{c_{⊥}}{r}$ . From the skew-symmetric definition in (6), we conclude that

$$l_{a}^{T}F_{a}=l_{a}^{T}\left({\hat{l}}_{a}a_{a}\right)=0,$$ (16)

The dynamics of a point-mass robot characterized by mass m and speed $v=|\dot{p}|$ is

$$F_{a}=m\frac{d\dot{p}}{dt}=m\left(\frac{dv}{dt}l_{a}+v\frac{dl_{a}}{dt}\right).$$ (17)

Substituting **F**_**a**_ into (16) and considering $l_{a}^{T}\frac{dl_{a}}{dt}=0$ , we conclude that

$$l_{a}^{T}F_{a}=m\frac{dv}{dt}=0.$$ (18)

The only solution to the previous equation is $\frac{dv}{dt}=0$ , which proves the claim that the robot’s speed is not affected by the force **F**_**a**_.

**FIGURE 4:**
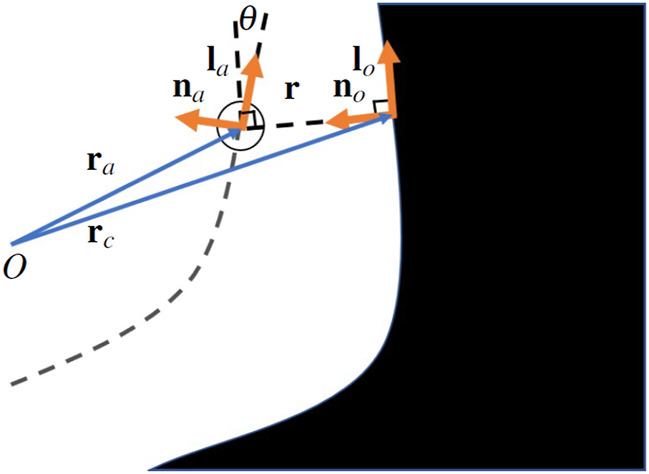
The scenario of a robot moving in the vicinity of a concave obstacle which has a continuous surface.

To prove obstacle avoidance, let us imagine an environment in which the obstacle has a concave continuous surface; this means that the surface is smooth with no discontinuity and each point on the obstacle surface can be described by a curvature parameter κ, which is a reciprocal of a radius of a surface. Suppose we have an obstacle whose closest point to the robot is located at vector **r**_c_ and the robot’s location is defined by vector **r**_a_ with respect to a static reference point O as illustrated in Figure 4. According to the Frenet-Serret formula in $R^{2}$ space (Millman and Parker, 1977), the surface of the obstacle can be described by the following equations

$$\begin{array}{c} \frac{\partial r_{c}}{\partial s}=l_{o},\frac{\partial l_{o}}{\partial s}=\kappa n_{o},\frac{\partial n_{o}}{\partial s}=-\kappa l_{o}, \end{array}$$ (19)

Where s represents curve segment, while **n**_o_ and **l**_o_ represent unit vectors whose direction is normal and perpendicular respectively to the closest obstacle surface as shown in Figure 4. *Via* the chain rule, we get the following set of equations

$$\begin{array}{c} {\dot{r}}_{c}=\frac{ds}{dt}l_{o},{\dot{l}}_{o}=\frac{ds}{dt}\kappa n_{o},{\dot{n}}_{o}=-\frac{ds}{dt}\kappa l_{o}. \end{array}$$ (20)

Since the robot’s speed is shown to be unaffected by the vector field, the robot’s motion can be described by

$$\begin{array}{c} {\dot{r}}_{a}=vl_{a},{\dot{l}}_{a}=\omega n_{a},{\dot{n}}_{a}=-\omega l_{a}, \end{array}$$ (21)

With $\omega =\frac{\left|F_{a}\right|}{mv}$ .

Lemma 6. For a concave obstacle which produces vector field **F**_**a**_ expressed in (15), the rate of angle **θ** between the robot’s direction and the obstacle surface (as illustrated in Figure 4) is given by

$$\dot{\theta }=\left(\frac{\kappa v}{1-\kappa r}-\frac{c_{⊥}}{mvr}\right)\cos\theta .$$ (22)

Proof from Figure 4, we can infer that

$$l_{o}^{T}n_{a}=\sin\theta .$$ (23)

Differentiating the equation with respect to time, we have

$${\dot{l}}_{o}^{T}n_{a}+l_{o}^{T}{\dot{n}}_{a}=\dot{\theta }\cos\theta .$$ (24)

Combining with 20, 21, we get

$$\frac{ds}{dt}\kappa n_{o}^{T}n_{a}+l_{o}^{T}\left(-\omega l_{a}\right)=\dot{\theta }\cos\theta .$$ (25)

Recalling that $n_{o}^{T}n_{a}=l_{o}^{T}l_{a}=\cos\theta$ , we can simplify the equation into

$$\dot{\theta }=\frac{ds}{dt}\kappa -\omega .$$ (26)

Using $|l_{a}\times \left(\frac{r_{o}}{r}\times l_{o⊥}\right)|=|l_{o⊥}|=\cos\theta$ , we can provide a simplified representation of ω as follows,

$$\omega =\frac{\left|F_{a}\right|}{mv}=\frac{c_{⊥}|l_{a}\times \left(\frac{r_{o}}{r}\times l_{o⊥}\right)|\frac{1}{r}}{mv}=\frac{c_{⊥}\cos\theta }{mvr}.$$ (27)

From Figure 4, we also get

$$r^{T}l_{o}=0.$$ (28)

Differentiating with respect to time, we get

$${\dot{r}}^{T}l_{o}+r^{T}{\dot{l}}_{o}=0.$$ (29)

Combining with 20, 21 and noting that $\dot{r}={\dot{r}}_{c}-{\dot{r}}_{a}$ , we get

$${\left(\frac{ds}{dt}l_{o}-vl_{a}\right)}^{T}l_{o}+\kappa \frac{ds}{dt}r^{T}n_{o}=0.$$ (30)

Combining the equation with the fact that $l_{a}^{T}l_{o}=\cos\theta$ and **r** ^T^ **n**_o_ = − r, we get

$$\frac{ds}{dt}=\frac{v\cos\theta }{1-\kappa r}.$$ (31)

Substituting the value of $\frac{ds}{dt}$ in (31) and ω in (27) to (26), we get

$$\dot{\theta }=\left(\frac{\kappa v}{1-\kappa r}-\frac{c_{⊥}}{mvr}\right)\cos\theta .$$ (32)

*Lemma 7. Force* ***F***_***a***_ *in* (15) *will guarantee collision avoidance with any continuous concave surface if the following conditions are satisfied:*

1. *the initial direction of* ***l***_*a*_ *is not in the direction of* ***r***_*o*_ *, and*
*2. the surface*’*s curvature* *κ* *and the robot*’*s distance to the surface* *r* *follows the following condition at all times*

$$\kappa <\frac{1}{r}.$$ (33)

Proof Using the chain rule of derivative, Eq. 22 can be modified into

$$\frac{d\theta }{dr}\dot{r}=\left(\frac{\kappa v}{1-\kappa r}-\frac{c_{⊥}}{mvr}\right)\cos\theta .$$ (34)

The robot’s velocity component in *y*-axis is equal to $-\dot{r}$ as follows

$$\dot{r}=-v\sin\theta .$$ (35)

Inserting the value of $\dot{r}$ from (35), we get

$$\int \frac{\sin\theta }{\cos\theta }d\theta =\int \left(-\frac{\kappa }{1-\kappa r}+\frac{c}{mv^{2}r}\right)dr.$$ (36)

Details on how to solve the integral are provided in the Appendix. The final equation has the following form

$$\left(1-\kappa r\right)r^{\frac{c}{mv^{2}}}\cos\theta =D,$$ (37)

Where D denotes the constant relating to the initial condition. We assume that the robot is in an initial condition described by:

1. r > 0, i.e. the robot does not touch the obstacle, and
2. (1 − κr) > 0, i.e. there is a single point on the obstacle surface that has the shortest distance to the robot.

Then, recalling the assumption that the initial angle $\theta \neq \pm \frac{\pi }{2}$ and the fact that $\theta \in \left(-\frac{\pi }{2},\frac{\pi }{2}\right)$ , we can conclude that cos θ > 0, which results in D > 0. In other words, in order that the robot-to-obstacle distance maintains the condition r > 0 (i.e. no contact with the obstacle surface), it is necessary for (1 − κr) > 0. This concludes the proof.

**FIGURE 5:**
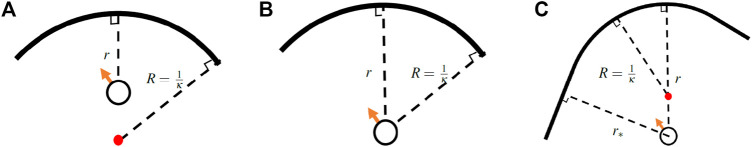
Three possibilities of a robot in the vicinity of a smooth concave obstacle: **(A)** the case where $\kappa <\frac{1}{r}$ , **(B)** the case where $\kappa =\frac{1}{r}$ , and **(C)** the case where $\kappa >\frac{1}{r}$ .

There are three possibilities regarding the relation between obstacle-to-robot distance r and curvature of the obstacle surface κ:

1. The first case, where $\kappa <\frac{1}{r}$ , describes the situation where there is only a single point on the obstacle surface closest to the robot, as illustrated in Figure 5A. Lemma 7 guarantees that in this condition, the algorithm makes sure that the robot will be able to avoid colliding with the obstacle.

2. The second case, $\kappa =\frac{1}{r}$ , refers to a condition where the robot is located at a distance r which is equal to the radius of curvature $\frac{1}{\kappa }$ of the obstacle surface, as illustrated in Figure 5B. This means that there are several points along the obstacle surface, each equidistant from the robot. In this case, with multiple closest points, the algorithm, as things stand, will not be able to guarantee collision avoidance.

3. The final case, where $\kappa >\frac{1}{r}$ , refers to a condition in which the point on the obstacle surface, where vector **r** is perpendicular to vector **l**_o_, is not the obstacle’s closest point to the robot, i.e. there are other points (at least one) along the obstacle surface which are closer to the robot, as illustrated in Figure 5C. Those other points are characterized by distance vector r_*_ (with |r_*_| < |**r**|), which is also perpendicular to vector **l**_o_. The actual point on the obstacle surface that is closest to the robot will then fall under one of the previous two cases.

Remark. From these three cases, we can conclude that Lemma 7 actually guarantees obstacle avoidance for any concave obstacle with a continuous surface and a single point that is closest to the robot. Cases with multiple closest points will be explored in Section 4.4.

### 4.3 Properties of Goal Convergence

For the chosen point-mass robot model (as described in Section 3), the control law for navigating the robot past obstacles towards the desired goal position is given by:

$$u=F_{g}+F_{o}.$$ (38)

The control law in (38) consists of an obstacle avoidance term **F**_**o**_ in (10) and a goal attraction term **F**_**g**_. For simplicity, in this section, we use a proportional-derivative (PD) controller for **F**_**g**_ with goal position **p**_**g**_ as the equilibrium point, represented as follows:

$$F_{g}=-K_{P}\left(p-p_{g}\right)-K_{D}\dot{p}.$$ (39)

Here, K_P_ and K_D_ are both positive constants.

To prove goal convergence, we assume that the robot is in an environment with small-sized non-maze obstacles such that the robot’s distance to the goal, when following the boundary of the obstacle surface, is never greater than its initial distance to the goal. By setting this constraint, we are assuming that the robot’s speed will never be zero as it follows the boundary. In this case, the control law in (38) is globally asymptotically stable.

Lemma 8. The control law in Eqs 38, 39 when applied to a point-like robot using the dynamic model described in (8), has a globally asymptotically stable equilibrium at the goal position **p**_**g**_. This assumes that the robot never stops while following the boundary, and that its direction of motion is not orthogonal to the closest obstacle surface.

Proof.The proof begins with the following Lyapunov function candidate

$$V=\frac{1}{2}{\dot{p}}^{T}\dot{p}+\frac{1}{2}K_{P}e^{T}e,$$ (40)

In which we define error vector as **e** = (**p** − **p**_**g**_). The rate of change of V is given by

$$\dot{V}={\dot{p}}^{T}\ddot{p}+K_{P}e^{T}\dot{e}.$$ (41)

Substituting (10), (38), (39) into (8) and considering that $\dot{e}=\dot{p}$ while, according to Lemma 1 and Lemma 5, ${\dot{p}}^{T}F_{o}={\dot{p}}^{T}F_{b}+{\dot{p}}^{T}F_{a}=0$ , the equation can be simplified into

$$\dot{V}=-K_{D}{\dot{p}}^{T}\dot{p}.$$ (42)

On this basis, the condition $\dot{V}\leq 0$ always holds including at **p** = **p**_**g**_ and $\dot{p}=0$ , which is the equilibrium point where $V=\dot{V}=0$ . Therefore, we can therefore conclude that this goal position is indeed a globally asymptotically stable equilibrium.

### 4.4 Extension for Special Cases Condition

Thus far, the proposed vector field **F**_**o**_ guarantees obstacle avoidance for: *1*) an arbitrary-shaped convex obstacle (irrespective of whether it has a continuous or discontinuous surface) and *2*) a concave obstacle provided that it has a continuous surface and a unique closest point at all times. The real world however can be uncooperative in this regard and is likely to present us with obstacles that do not adhere to these requisite constraints. There are objects with concave sharp corners or with surfaces whose curvature κ changes abruptly. We therefore need to consider situations in which we may not have a unique point on the obstacle’s surface that is closest to the robot. To mitigate this issue, we propose an extension to our original algorithm.

**FIGURE 6:**
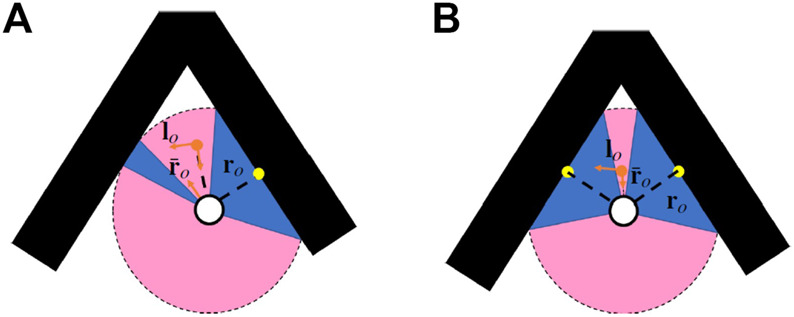
The averaging technique employed to post-process the sensory information can be used to solve the problem of a concave sharp corner where **(A)** the curvature surface is discontinuous and **(B)** there are non-unique closest points.

Previously, we had assumed that the robot identifies the closest point on the obstacle surface from its sensor readings by simply choosing the point with the smallest distance value out of a selection of readings. In the revised scenario, the robot obtains this value by computing the average over several sensed distances. The robot chooses a number of sensed distances from current sensor readings which are smaller than a specified threshold δr. It then calculates the average distance value from these readings, $\left({\bar{r}}_{o}\right)$ , which it uses for subsequent calculations. For a concave obstacle, this distance value $\left({\bar{r}}_{o}\right)$ will always be smaller in magnitude than the closest sensed position, i.e. $\left|{\bar{r}}_{o}|<|r_{o}\right|$ , as illustrated in Figure 6, and so we use this value as the new closest point. For a convex obstacle, however, the condition is reversed, i.e. $\left|{\bar{r}}_{o}|\geq |r_{o}\right|$ , and we can therefore revert to the standard position data **r**_o_ as the vector describing the closest point between the obstacle and the robot.

The robot is now able to navigate around both convex and non-convex obstacles, even when surfaces are not continuous (such as a sharp corners, as in Figure 6). This holds true as long as the robot’s initial direction is not entirely in line with the vector connecting the robot and the closest obstacle point $\left(\theta \neq \frac{\pi }{2}\right)$ . This configuration could theoretically cause the artificial current **l**_o_, a projection of the robot’s speed **l**_a_ towards the obstacle surface, to drop to zero. The obstacle would then produce no magnetic field to repel the advancing robot. From a practical perspective, this would be unlikely to happen as noise in the sensor’s measurements would naturally yield a non-zero angle (even if it is a small one) between the two vectors **l**_a_ and **r**_o_. To reduce the effect of this problem, we use only the unit vector of the previously proposed artificial current when the magnitude of the standard artificial current is less than a positive constant ϵ. Another special case is when the environment consists of a large-size obstacle such that the robot’s distance to the goal when circumnavigating the obstacle could be larger than its initial distance to the goal. Using only the PD control in (39), in this type of environment, the robot could arrive at a zero-speed situation, i.e. when the robot loses most of its kinetic energy due to the dissipation term in the attractive field. To overcome this problem, we add a goal relaxation (GR) mechanism, having the property of decreasing the goal attraction **F**_**g**_ when the robot is close to the surface of the obstacle, while the goal is still occluded by the obstacle, and, vice versa, to increase the goal attraction when the obstacle stops obstructing the desired position. Please refer to (Ataka et al., 2018a) for more details on strategies for the last two special cases.

## 5 Implementation

We implemented our magnetic-field-inspired navigation algorithm to steer a dynamic model of a simulated point-like robot using the control law described in (38). We first used PD control as described in (39) as a goal attraction term **F**_**g**_ to guide the robot towards its desired target. Constants K_P_ and K_D_ are determined *via* trial and error, given the maximum actuating capability of the robot.

The second term **F**_**o**_ of the control law (38) relates to obstacle avoidance, and consists of a boundary-following vector field **F**_**b**_ described in 11–13 and a collision-avoidance vector field **F**_**a**_ described in 14, 15. In our algorithm implementation, the boundary-following vector field is only used when the robot’s closest distance to the obstacle falls below a specified limit r_l_, and the collision-avoidance vector field is only used when the robot get closer still, its threshold level being the distance r_la_.

$$F_{o}=\left\{\begin{array}{cc} F_{b}+F_{a}\; & \text{if }|r_{o}|<r_{la}\\ F_{b}\; & \text{if }|r_{o}|\geq r_{la}\text{ and }|r_{o}|<r_{l}\\ 0\; & \text{if }|r_{o}|\geq r_{l} \end{array}\right..$$ (43)

The PD control law referred to in (39), however, does not guarantee constant robotic speed. A scenario could occur in which attraction to the goal causes the robot’s speed v to increase when under the influence of a nearby obstacle. To prevent this from occurring, we introduced a geometric control term as an alternative to guide the robot towards the goal, loosely based on the work described in (Bullo and Murray, 1995) for SO(3). This control term has an interesting property: it is shown to globally asymptotically guide the robot’s movement (toward the goal position **p**_g_) without affecting the robot’s speed. A full description of geometric control implementation is given in (Ataka et al., 2018b) for mobile robot and in (Ataka et al., 2019) for quadcopter robot.

In order to implement the algorithm on a 7-DOFs Baxter arm, we applied the vector field in the task space of the robot and used the dynamic model of the robot to produce the joint accelerations. The vector field used to guide the point-like robot in (38) is redeployed to guide the tip of the Baxter arm towards its goal while avoiding obstacles along the way. A repulsive field is also applied to the body of the manipulator to mitigate potential impact, but to ensure that it doesn’t affect the behaviour of the manipulator tip, we apply the torque in the null space of the Jacobian as described in (Brock and Khatib, 2002).

## 6 Results and Analysis

To analyze and evaluate the performance of the proposed algorithm, we present our results from both simulations and an experimental study. Our magnetic-field-inspired navigation system was applied to a point-like robot model and tested in several scenarios in $R^{2}$ and $R^{3}$ . The algorithm was also tested as a guiding method in a Baxter manipulator tip, a 7-DOFs industrial robot platform. The task was for the tip to avoid obstacles in $R^{3}$ , while the manipulator body itself was prevented from collisions by the application of a repulsive potential. We assume that the robot is only able to detect the surrounding environment within a range r_s_ = r_l_ = 0.3 m in all directions relative to its position. Some obstacles are generated as point clouds from a 3D model of the real environment; others are specifically designed to highlight the advantages of our algorithm when compared to other methods. This comparative study includes our magnetic-field-inspired (MFI) navigation system (with both the PD and geometric controllers) alongside several other navigation methods presented in the literature - specifically the standard APF (Khatib, 1985), the circular field (CF) (Haddadin et al., 2011), and the gyroscopic force (GF) (Sabattini et al., 2013). These methods were chosen as they share the same properties as the proposed algorithm, i.e. they are all reactive navigation methods, able to operate without the need for prior environmental mapping and are capable of operating in 3D environments populated with arbitrarily shaped obstacles. The system comparisons focus on trajectory covered and time needed to reach the goal while avoiding obstacles en route. All our simulations and experiments use the Robot Operating System (ROS) as a programming framework (Quigley et al., 2009). The parameter values of the algorithm are summarised in Table 1. These parameters are retrieved from trial and error. We choose the parameters which produce the most desirable trajectory in terms of the path length and the time required to cover the path.

**TABLE 1:** List of parameter values used in the algorithm.

Param	Point-like robot	Baxter simulation	Baxter experiment
*c*	10	4	20
*c_⊥_*	20	0.08	0.08
*K_P_*	0.04	20	5
*K_D_*	0.5	10	10
*r_l_*	3 m	0.3 m	0.3 m
*r_la_*	2 m	0.2 m	0.2 m
*ϵ*	3 × 10^−6^	0.05	0.05

### 6.1 Simulation Results for Point-like Robot

**FIGURE 7:**
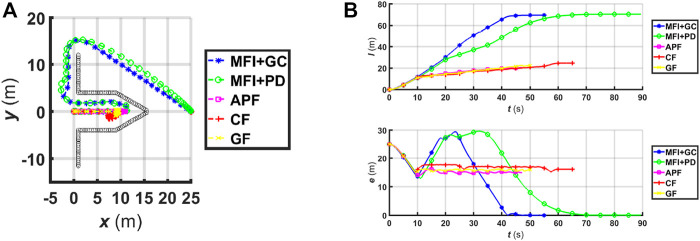
**(A)** The robot’s trajectory drawn in dashed lines. The environment consists of a sharp corner obstacle. **(B)** The plot of the covered trajectory l(t) and the positional error e(t) as function of time for 2D sharp corner environments.

In the first simulation, depicted in Figure 7, our MFI algorithm is implemented in a point-like robot moving in a 2D plane containing a sharp corner. In this scenario, Figure 7A, we see that it is able to guide the robot towards its goal, using either geometric (MFI+GC) or PD control (MFI+PD). The other three algorithms, namely the APF, CF, and GF all fail to reach the goal. The APF fails due to the cancelling out of the attractive terms towards the goal by the repulsive field from the obstacle. The CF and GF, on the other hand, fail due to the zero speed condition caused by the attractive field towards the goal as seen from the saturated behaviour of the robot trajectory l(t) and positional error e(t) in Figure 7B. The reason this issue does not arise with our algorithm (whether using PD or geometric control) is that the collision-avoidance vector field **F**_**a**_ in (15) has the property of repelling the robot without affecting its speed. This is a major advantage over the CF and GF methods. In this 2D scenario, we also observe how the averaging technique described in Section 4.4 helps the robot avoid the sharp corner despite it having a number of non-unique points of closest distance to the robot.

**FIGURE 8:**
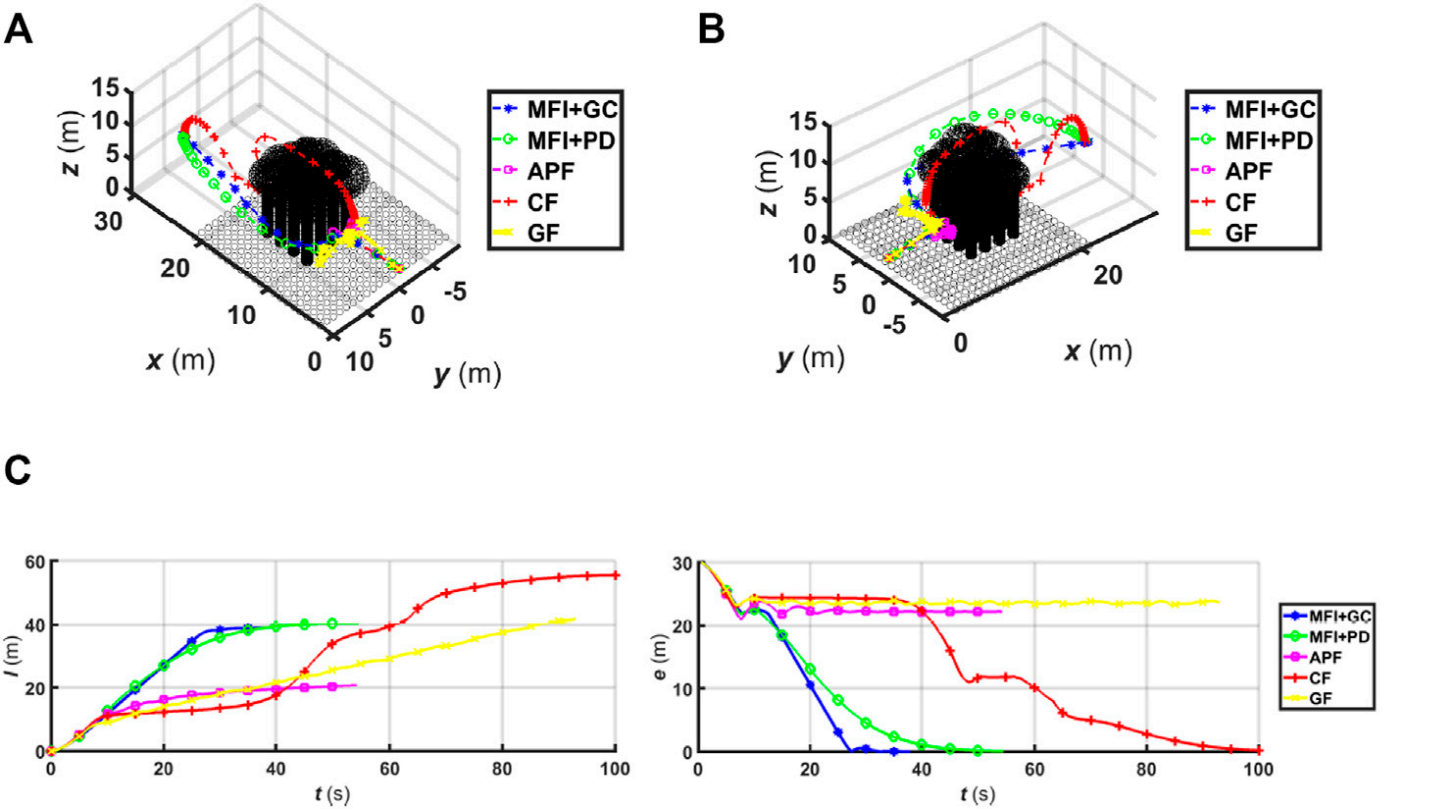
The robot’s trajectory drawn in dashed lines from two different perspectives (**(A**,**B)**). The environment consists of a forest-like environment. **(C)** The plot of the covered trajectory l(t) and the positional error e(t) as function of time for 3D forest environments.

To test the methods in a more realistic 3D scenario, we designed a forest-like environment consisting of cylinders and spheres that mimic trunks and leaves as shown in Figures 8A,B. As before, we can see that the proposed algorithm (whether under geometric or PD control) is able to successfully navigate the robot amidst arbitrarily shaped obstacles towards the desired goal position. The APF and GF methods both fail to circumvent the trees in this environment due to the effect of local minima (in the case of APF) and of the zero-speed problem (in the case of GF). The CF method is able to guide the robot to its goal, but does so at the expense of a longer trajectory and convergence time as shown in Figure 8C despite its knowledge of the obstacle’s centre point.

**FIGURE 9:**
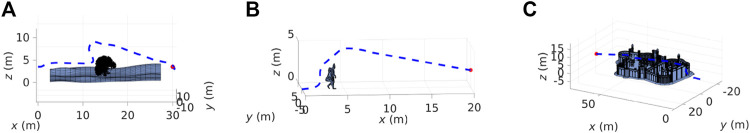
The robot’s trajectory using the magnetic-field-inspired navigation with geometric control in different environments populated with **(A)** a tree, **(B)** a human, and **(C)** a castle.

To further demonstrate the ability of the proposed algorithm in handling realistic scenarios, Figure 9 shows the trajectory of the robot in more challenging environments that incorporate a tree (Figure 9A), a human (Figure 9B), and a castle (Figure 9C). In all these scenarios, we see that the proposed MFI algorithm is able to navigate the robot through complex environments that are cluttered with a range of obstacles differing in shape, convexity and surface continuity, while relying only on a range sensor and without any pre-knowledge of the environment. This last point is crucial to the algorithms potential application in which environment maps are unavailable such as in post-disaster sites or open spaces with unpredictable landscapes.

### 6.2 Experimental Results Using Baxter Arm

**FIGURE 10:**
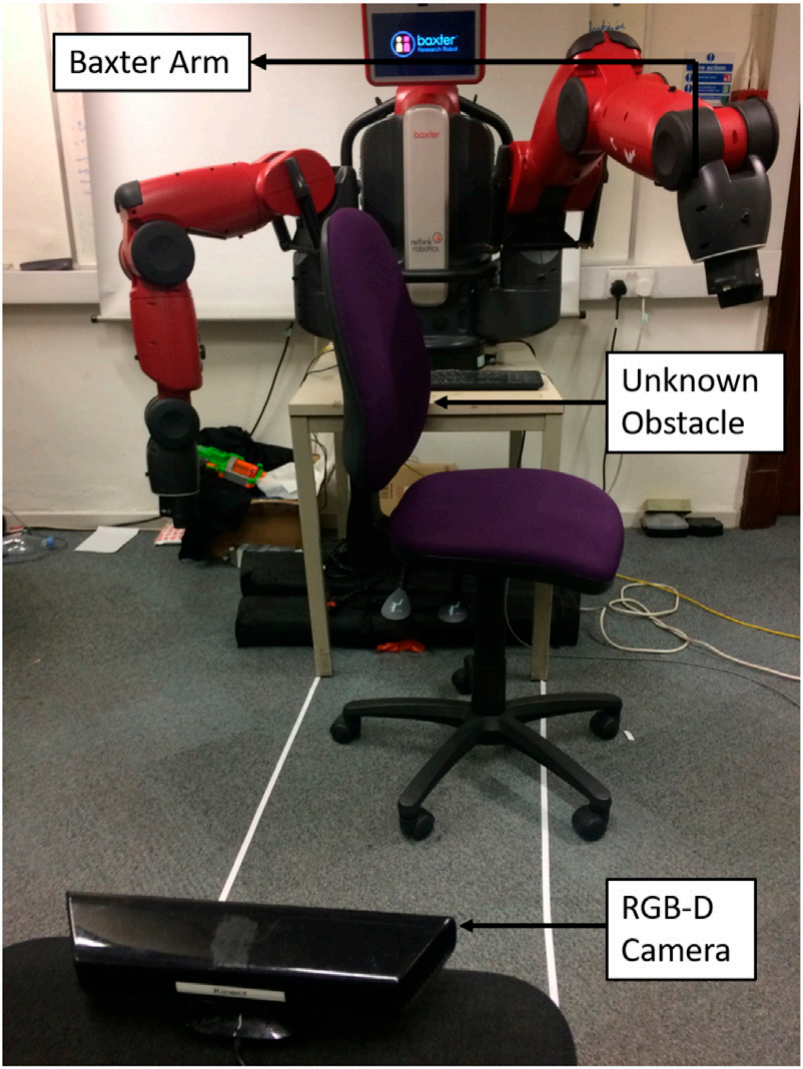
The figure depicts experimental setup consisting of a seven DOFs Baxter manipulator. The environment consists of a chair detected by RGB-D camera.

**FIGURE 11:**
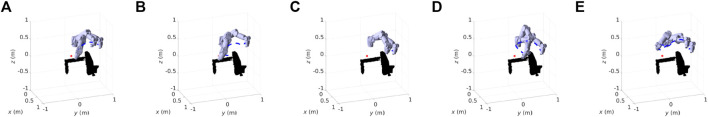
The motion of the Baxter arm in a real scenario with environment consisting of black chair and table (drawn in black) using **(A)** the MFI algorithm with geometric control, **(B)** the MFI algorithm with PD control, **(C)** standard APF method, **(D)** CF method and **(E)** GF method, respectively. The dashed lines are the tip’s trajectory.

In our experimental implementation, the proposed algorithm’s guidance capabilities were tested to guide the tip of a 7-DOFs Baxter Arm as depicted in Figure 10. The environment consisted of a chair whose location was unknown to the robot at the outset. The Kinect RGB-D camera was used to detect the position of the obstacle and to feed that information to the robot only once the tip of the robot’s arm got closer to the obstacle than limit distance r_s_. Figure 11 shows the movement of the Baxter arm using the proposed MFI with geometric control (Figure 11A), MFI with PD control (Figure 11B), the APF method (Figure 11C), the CF method (Figure 11D), and the GF method (Figure 11E). We see that the proposed algorithm with geometric control (Figure 11A) is able to guide the robot towards the target while smoothly avoiding the obstacle. The APF method (Figure 11E) results in local minima issues, while the other algorithms produce non-smooth robotic arm movements.

**FIGURE 12:**
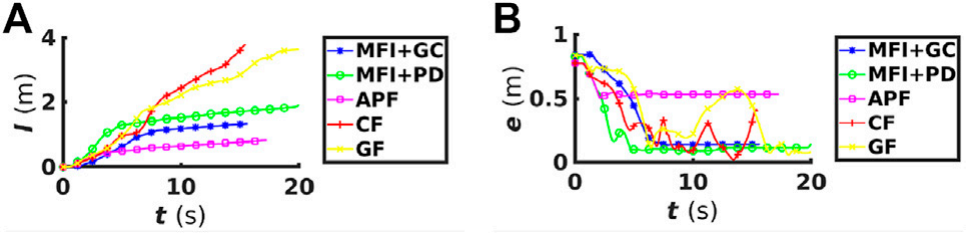
**(A)** The plot of the trajectory covered by the Baxter’s tip l(t) and **(B)** the position error e(t) as function of time for the case of real environment with chair as an obstacle.

To further highlight the superiority of the proposed algorithm, we present the performance comparison between the five methods in Figure 12. Bar the APF method, which fails to guide the robot to the goal, the others show similar performance to our MFI algorithm in terms of the time the robot needs to reach the goal - as evidenced by the plot of error e(t). It is noted, however, that the CF and GF algorithms fail to fluidly guide the tip to its goal in the real scenario - as evident from the unstable value of error e(t) in Figure 12. This is due to the non-existence of the collision-avoidance vector field in the case of CF and the use of the repulsive term causing the tip to be repelled too far from its path in the case of GF. In terms of the trajectory taken by the tip, we can see that the proposed algorithm with geometric control and the same with PD control take the shortest and second shortest paths, respectively.

### 6.3 Discussion

Table 2 summarizes the simulation and experimental results, comparing the artificial potential field (APF) method, the circular field (CF) method, the gyroscopic force (GF) method, and our magnetic-field-inspired (MFI) algorithm both with PD control and with geometric control (GC). The comparisons among these navigation methods are conducted in terms of their abilities in obstacle avoidance, goal reaching, and path quality. The latter parameter is judged by the length of trajectory l(t) and convergence time, i.e. a time needed for the distance error e(t) to fall below a specified value e_b_. When an algorithm fails to navigate the robot to its desired target, these variables are not taken into account. We chose the distance threshold parameter to be set at e_b_ = 0.05r_gi_ in a simulation scenario in which r_gi_ denotes the initial distance between the robot and its target at the outset, but opted for e_b_ = 0.2 m for the experimental setup, taking into account the steady-state positional error of the Baxter’s tip.

**TABLE 2:** Summary of Results.

Robot	Obstacle	Algorithm	Success	Covered path (m)	Time (s)
Point-Like Robot 2D	Sharp Corner	APF	×	—	—
		CF	×	—	—
		GF	×	—	—
		MFI + PD	*✓*	70.51	61.85
		MFI + GC	*✓*	69.64	41.03
Point-Like Robot 3D	Forest	APF	×	—	—
		CF	*✓*	55.80	86.13
		GF	×	—	—
		MFI + PD	*✓*	40.46	37.95
		MFI + GC	*✓*	38.91	26.08
Baxter Experiment	Chair	APF	×	—	—
		CF	*✓*	3.80	6.58
		GF	*✓*	3.69	16.3
		MFI + PD	*✓*	2.03	3.08
		MFI + GC	*✓*	1.35	6.21

Table 2 indicates that in both the simulation and the experimental setup, our proposed reactive magnetic-inspired-field navigation algorithm outperforms other navigation methods in almost every tested scenario. This is of particular note in regard to two aspects: the ability of the algorithm to successfully navigate the robot to its desired target amidst an obstacle-laden environment and its fast convergence time of the positional error. We see that the proposed MFI algorithms, both with PD control or geometric control, are successful in every scenario. Our algorithms are significantly more successful in environments with concave obstacles, such as those with sharp corners. They also outperform others in environments consisting of arbitrarily shaped obstacles, such as a forest configuration. In all simulation scenarios, the proposed method with GC control invariably comes first in terms of its convergence speed. This is principally due to the fact that the whole vector field (both the goal attraction and the obstacle avoidance terms) does not influence the robot’s speed, other than at the very beginning and the final part of the robot’s motion. Indeed it is this constant speed that facilitates obstacle avoidance, enabling fast and fluid movement around the boundary of any obstacle. In addition to convergence speed, the proposed algorithms also achieve the shortest trajectory - both in the simulation as well as the experimental setup.

Most importantly, our MFI navigation algorithm does not rely on prior knowledge of the environment, such as obstacle geometry or location, as is the case with other magnetic-field-inspired navigation methods (Singh et al., 1996; Haddadin et al., 2011). Our algorithm only requires information from a local sensor able to measure the spatial distance to nearby obstacles along with information regarding the robot’s speed. Compared to other gyroscopic-based navigation methods, the proposed algorithm is superior since it can be applied not only in 2D environments, but also in 3D environments, with convex obstacles or non-maze concave obstacles, including those obstacles with sharp corners. In terms of computational burden, our algorithm (summarised in Eq. 11 and Eq. 15) only uses simple matrix multiplication process. The same process is also employed by gyroscopic-based methods. In conclusion, our algorithm achieves better performance in general despite having similar computational burden to other reactive navigation methods.

## 7 Conclusion

This paper presents and examines a reactive navigation method that can guide a robot toward a target position within a 3D environment that is laden with arbitrarily shaped convex and non-maze concave obstacles. Drawing inspiration from magnetic field laws, we produce an algorithm capable of steering a robot away from and around obstacles that lie en route to the target. It is demonstrably superior than the standard APF method, as it is free from local minima in environments consisting of both convex and non-maze concave obstacles. Compared to other magnetic-field-inspired navigation methods, ours has a further key advantage in that requires no prior knowledge of the environment, in terms of geometry or obstacle location. The algorithm is shown to successfully navigate a point-like robot model in both $R^{2}$ and $R^{3}$ and a 7-DOF Baxter arm towards a specified goal in the presence of previously unknown obstacles. The results show that our navigation algorithm has the potential to be used in a wide range of devices, such as flying robots, underwater robots, soft continuum manipulators, and even in swarming multi-robot systems. Future work will consider the limitations of robotic actuators and make in-roads into using such systems in unknown maze-like environment and unknown environments with dynamics obstacles.

## Data Availability

The raw data supporting the conclusions of this article will be made available by the authors, without undue reservation.

## Appendix: Solving $\int \frac{\sin\theta }{\cos\theta }d\theta =\int \left(-\frac{\kappa }{1-\kappa r}+\frac{c}{mv^{2}r}\right)dr$

Assuming u = cos *θ*, we have *du* = − sin *θdθ*. Hence, the left side of the equation can be transformed into

$$-\int \frac{1}{u}du=-\ln u+E,$$

Where E denotes an integration constant. Defining *v* = (1 − *κr*), *we* get *dv = − κdr*. Thus, the right side of the equation can be simplified into

$$\int \frac{1}{v}dv+\frac{c}{mv^{2}}\int \frac{1}{r}dr=\ln v+\ln r^{\frac{c}{mv^{2}}}+F,$$

Where *F* denotes an integration constant. Combining the two terms together, we get

$$\ln\left(vur^{\frac{c}{mv^{2}}}\right)=G,$$

$$vur^{\frac{c}{mv^{2}}}=e^{G}=D,$$

Where *G* and *D* are constants. Substituting the value of *u* and *v*, we arrive at

$$\left(1-\kappa r\right)r^{\frac{c}{mv^{2}}}\cos\theta =D,$$

And hence, conclude the derivation.
